# Health inequality in Britain before 1750

**DOI:** 10.1016/j.ssmph.2021.100957

**Published:** 2021-11-16

**Authors:** Ellen J. Kendall, Alex T. Brown, Tim Doran, Rebecca Gowland, Richard Cookson

**Affiliations:** a Department of Archaeology, Durham University, Durham, UK; b Department of History, Durham University, Durham, UK; c Department of Health Sciences, University of York, Heslington, York, UK; d Centre for Health Economics, University of York, Heslington, York, UK

**Keywords:** Life expectancy, Early modern England, Childhood mortality, Social inequality, Historical demography

## Abstract

**Background:**

This study examines the claim that social inequality in health in European populations was absent prior to 1750. This claim is primarily based on comparisons of life expectancy at birth in England between general and ducal (elite aristocrat) social classes from the 1550s to the 1870s.

**Methods:**

We examined historic childhood mortality trends among the English ducal class and the general population, based on previously published data. We compared mid-childhood to adolescent mortality (age 5–14) and early-childhood mortality (age 0–4) between the ducal class and the general population from the 17^th^ to 19^th^ centuries.

**Results:**

Prior to 1750, ducal early-childhood mortality was higher than the general population. However, mid-childhood to adolescent mortality was lower among the ducal class than the general population in all observed periods for boys, and almost all periods for girls. Among the ducal class, but not the general population, there was a sharp decline in early-childhood mortality around the 1750s which may partly explain the divergent trends in overall life expectancy at birth.

**Conclusion:**

Health inequality between the ducal class and general population was present in England from the 16^th^ to mid-18^th^ centuries, with disadvantages in mortality for ducal children in infancy and early childhood, but survival advantages in mid-childhood and adolescence. These opposing effects are obscured in life expectancy at birth data. Relatively high early-childhood mortality among ducal families before 1750 likely resulted from short birth intervals and harmful infant feeding practices during this time.

## Highlights

• We re-examine the claim that social inequality in health was absent in Britain before 1750.
• This claim is based on life expectancy at birth among ducal and general populations.
• We found consistently “pro-ducal” mortality gaps from age 5 to 14.
• “Anti-ducal” under-5 mortality before 1750 may relate to short ducal birth intervals and feeding practices.
• Social inequality in health did exist before 1750, but varied by age group.

## 1 Introduction

Reducing unfair differences in health between more and less socially advantaged groups – known variously as “health inequalities” in the UK, “health disparities” in the USA, and “health inequities” by the World Health Organisation – is a major priority for public policymakers in the 21^st^ century. Health inequalities originate in social inequalities and can be further exacerbated by social, economic, environmental, or public health crises, such as the COVID-19 pandemic (Cash-Gibson et al., 2021). Identifying the root causes of health inequalities, and associated strategies for decreasing them, has been a topic of interest to medical historians and advocates of public health since the 19^th^ century. Correctly attributing the causes of historic health inequality remains a subject of great importance, as it has implications for informing policy in the present.

Several scholars have pinpointed the mid-18^th^ century as a watershed moment for health inequality in Europe, arguing that substantial differences in health outcomes relating to social inequality first emerged at that time. This claim has been based on historical demographic data on trends in life expectancy at birth, comparing general and ducal (elite aristocrat) classes, from which causal inferences have been drawn (Davey Smith, 2003; Deaton, 2013; Lindström & Davey Smith, 2019; Livi-Bacci & Croft-Murray Trans, 1991). However, other scholars have argued that European health inequality existed prior to modernity (e.g., Mackenbach, 1995; Pitts & Griffin, 2012; Robb et al., 2021). In this paper we re-examine the evidence for the assertion that health inequality first emerged after 1750, by taking a closer look at age-specific mortality trends and their determinants.

In the context of mid-20^th^ century scientific optimism, the physician and medical historian Thomas McKeown proposed that the causes of decreasing mortality, population growth, and improved health in high-income countries from the late 18^th^ century were not primarily attributable to advances in modern medicine, but to improved living standards (McKeown et al., 1972; McKeown & Brown, 1955). While McKeown acknowledged the contribution of medical innovations such as antibiotics and vaccination in reducing mortality, he demonstrated that long-term decreases in population mortality began well before their development. These proposals were controversial at the time they were made and continue to stir disagreement among historical demographers and epidemiologists (Colgrove, 2002; Grundy, 2005; Szreter, 2000). While a more extensive review of the literature is beyond the scope of this short paper, it is clear that wider social determinants of health include not only sanitation and nutrition, but a much broader range of economic and social circumstances that influence physiological, socio-emotional and cognitive development in childhood, and these continue to have cumulative impacts on health across the life course and inter-generationally (Almond et al., 2018; Barker, 2012; Link & Phelan, 2002; Marmot et al., 2008).

More recently, the economist Angus Deaton has questioned the role of social determinants in historic health inequalities (Deaton, 2013), claiming that advances in medical technology from the 1750s enabled social elites to start drawing away from the general population in terms of health and longevity. Referencing a paper by Harris (2004) comparing British ducal and English average life expectancy between the 16^th^ and 19^th^ centuries, Deaton argued that “kings, queens and dukes were always richer and more powerful than the population at large and would surely have used their money and power to lengthen their lives, but before 1750 they had no effective way of doing so …” (Deaton, 2013, p. 254). Suggesting that social inequalities in health did not exist before the mid-18^th^ century, he argued that the emergence of health inequality was driven by inequality of access to increasingly effective medical technology (e.g., variolation or medicinal treatments such as cinchona bark for malaria) rather than trends in wider social determinants of health. Deaton is not the first to call the existence of pre-modern health inequalities into doubt, or even the first to do so using Hollingsworth's peerage data (e.g., Antonovsky, 1967; Woods & Williams, 1995). Other researchers in epidemiology and demography have also expressed scepticism about the existence of health inequalities prior to the 18^th^ century, based on similar comparisons of class-based mortality data in England and sub-national areas of Sweden and Quebec (Edvinsson & Broström, 2017; Gagnon et al., 2011; Harris, 2008).

## 2 Ducal and wider English population life expectancy at birth: the data

**Fig. 1:**
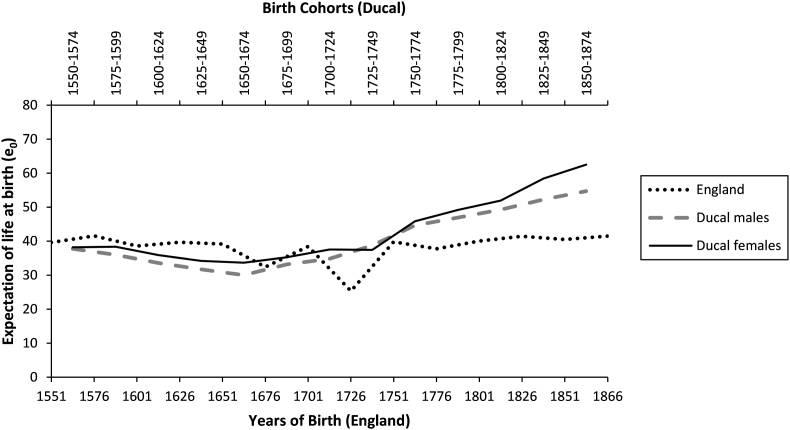
Life expectancy at birth (e0) from the 1550s–1870s, comparing ducal males and females (data from Hollingsworth (1977), Table 3, p. 328) with the general population of England (data from Wrigley et al. (1997), Table A9.1, p. 614).

The historic data on life expectancy at birth (e0) for British ducal families and the general English population drawn upon by researchers, including Harris (2004), are largely derived from work by Hollingsworth (1957, 1977) on the legitimate offspring of kings, queens, dukes and duchesses (ducal families) and Wrigley et al. (1989, 1997)'s reconstitution of English families from parish records. These data are both potentially problematic. Whilst being well-documented, the aristocracy represented an extreme element of the wealthy classes, facing elevated risk of violent and premature death in adulthood through executions and power struggles (Hollingsworth, 1965), and childhood mortality due to the prevalence of consanguineous marriage. Smith and Oeppen (2006) additionally suggest that Hollingsworth's data underestimates aristocratic mortality. Similarly, English parish data suffer significant biases relating to the non-standardization of source material prior to civil registration and the introduction of the census, as mortality in family reconstitution is based on families remaining within their parish, and crude aggregation of heterogeneous classes. English society in the late medieval and early modern period was highly stratified, with significant differences in living standards across classes. While acknowledging the problems inherent to ducal and family reconstitution data, for the sake of consistency we will interrogate these datasets to reassess the claim that health inequality did not exist before 1750. Fig. 1 reproduces data from Hollingsworth (1977, Table 3, p. 328) and Wrigley et al. (1997, Table A9.1, p. 614), with a visible gap in life expectancy between ducal and non-ducal populations emerging from around the middle of the 18^th^ century.

Without further disaggregation or historical investigation of the social context, these data appear to suggest that social inequality in health only emerged in the 18^th^ century. Life expectancy is similar between British ducal males, females, and the general English population prior to 1750. After 1750, not only do disparities emerge between ducal and general life expectancy data, but also *within* aristocratic data, as ducal females gain a growing advantage over ducal males. However, disaggregating data by age suggests that life expectancy at birth may be a misleading summary statistic in this particular historical context, and that health inequalities may have existed before 1750.

## 3 Differential mortality before 1750: a closer look

**Fig. 2:**
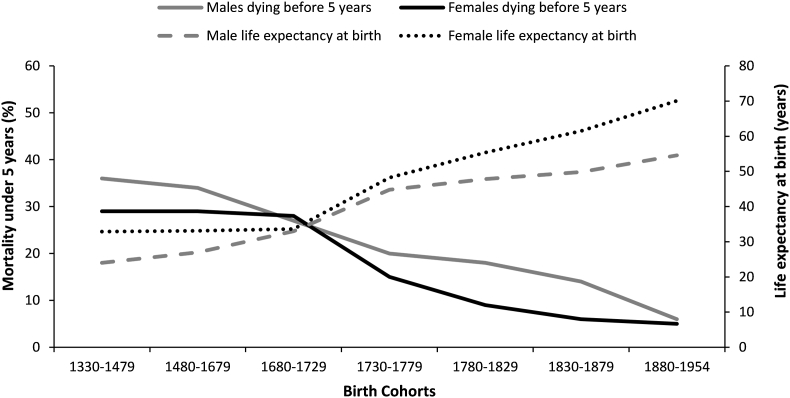
Ducal life expectancy at birth and child mortality (deaths under 5 years) from the 14^th^-19^th^ centuries by sex (life expectancy data from Hollingsworth (1957), p.8; data for deaths under 5 years from ibid, p. 9; the unevenly spaced cohort intervals are original to the Hollingsworth data).

From the 18^th^ to mid-20^th^ centuries, gains in life expectancy at birth are known to have been largely (though not entirely) a function of decreasing under-5 mortality, including both infant mortality (deaths following a live birth and before the age of 1 year) and child mortality (deaths occurring between the first and fifth birthday) (Aburto et al., 2020). While improvements in 18^th^ century adult ducal mortality are also reported by Hollingsworth (1977), the nature of life expectancy at birth is such that early deaths exert a disproportionate effect, relative to later mortality. Accordingly, it is reasonable to question how far the gains in ducal life expectancy at birth might be attributable to changes in under-5 mortality. Data in Fig. 2 for ducal males and females show an inverse relationship between life expectancy at birth (Hollingsworth, 1957, p. 8) and the percentage of children of either sex dying before the age of 5 years (Hollingsworth, 1957, p. 9). A substantial drop in under-5s deaths and rise in total life expectancy is observable for ducal females in the 1730–1779 birth cohort data. For ducal males, under-5 mortality and life expectancy undergo a similarly sharp change, but starting earlier, in the later 17^th^ and early 18^th^ century birth cohort.

**Fig. 3:**
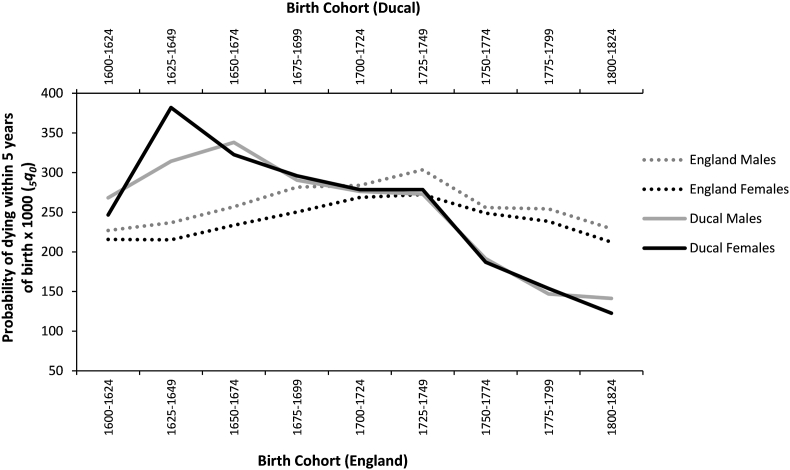
Under-5 mortality (0–4 years) risk from the 17^th^-19^th^ centuries, for ducal and wider English population by sex (data for ducal deaths from Hollingsworth (1964), p.54-55; data for English children calculated from 1q0 and 4q1 in Wrigley et al. (1997), p. 296 using WHO method for CME (2013)).

Comparisons of under-5 mortality risk (5q0) for ducal and general English populations between the 17^th^ and 19^th^ centuries are given in Fig. 3 (data for ducal deaths from Hollingsworth (1964), p.54-55; data for English children calculated from 1q0 and 4q1 in Wrigley et al. (1997), p. 296 using WHO method for CME (2013)). Prior to the mid-18^th^ century, ducal children's chances of dying before the age of 5 were similar or substantially higher than that of the general population of England, with ducal mortality being highest in the mid-17^th^ century.

**Fig. 4:**
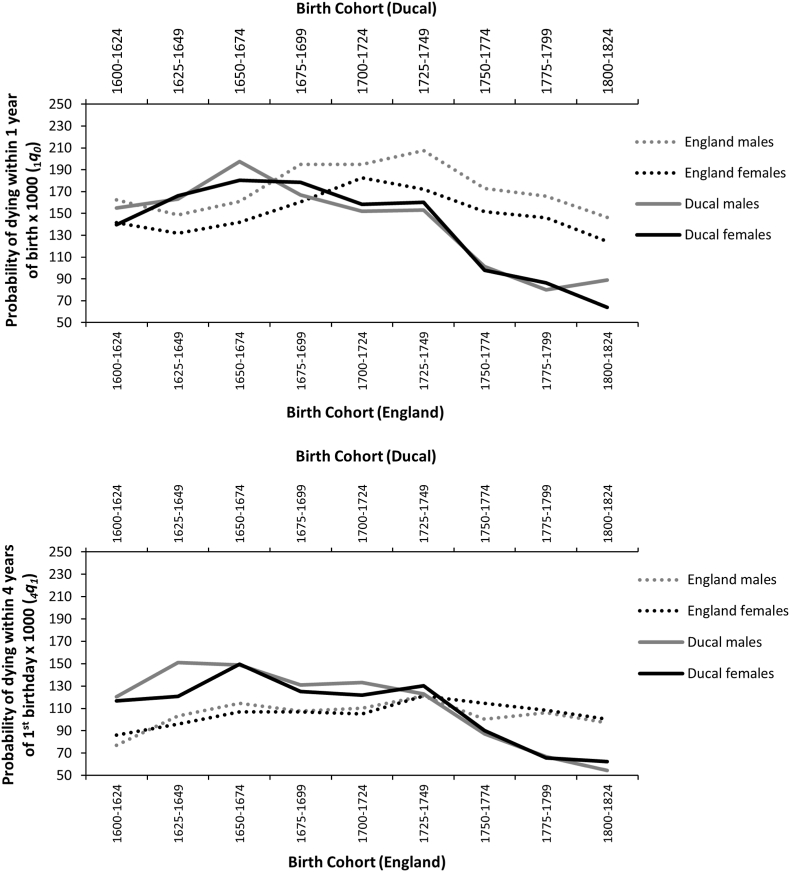
Infant (top panel) and child mortality (bottom panel) risk for ducal and wider English population by sex from the 17^th^-19^th^ centuries (data for ducal deaths from Hollingsworth (1977), p.327; data for English children in Wrigley et al. (1997), p. 296).

Fig. 4 shows the constituent elements of under-5 mortality, divided into infant (under 1 year) and childhood (1–4 years) mortality, to provide further insight into the nature of change within ducal child mortality patterns (data for ducal deaths from Hollingsworth (1977), p.327; data for English children in Wrigley et al. (1997), p. 296). Ducal infant mortality is similar to general English infant mortality prior to the mid-18^th^ century, when ducal infant mortality dropped steeply. In contrast, ducal child mortality is consistently *higher* than that of non-elite English children until around 1750, when a similarly substantial decrease in mortality occurred, and ducal mortality dropped well below English child mortality. In all periods, English infant mortality risk is higher than English child mortality. However, in the ducal population infant and child mortality are similar prior to 1750, without a substantial reduction in risk occurring with age. As it would not be expected that exposure to infectious disease or accident would be higher in elite environments, where aristocratic children were more likely to receive the environmental benefits of wealth and in-home care by a nurse (Newton, 2011), this may be expressive of relatively high intrinsic frailty and vulnerability to disease in many ducal children.

Thus far, we have not found evidence against the claim that improved medical technology was the main driver of diverging life expectancy between rich and poor, as childhood mortality did not begin to decrease among the general population until at least the mid-19^th^ century (Woods et al., 1989). However, these data patterns do suggest that gains in life expectancy (e0) in the mid-18^th^ century may be largely attributable to decreases in ducal child mortality (age 1 to 4). Furthermore, while deaths before the age of 5 years have an outsized impact on life expectancy, they do not in themselves provide a reliable indicator of population health or mortality across the rest of the life course (Murray, 1988). Unfortunately, the nature of published historic data makes like-for-like adult life expectancy comparisons difficult. Mortality risk by age is sex-specific (Wang et al., 2012) and, in contrast to Hollingsworth's aristocratic data, English adult data reported by Wrigley et al. (1997) is sex-aggregated. Comparative sex-specific historical mortality data from middle childhood and adolescence may thus provide a clearer picture of health inequalities. Mortality risk is typically lowest in this age group (Chamberlain, 2006; Gage, 1989), and non-status related factors associated with mortality in infancy and adulthood are avoided.

**Fig. 5:**
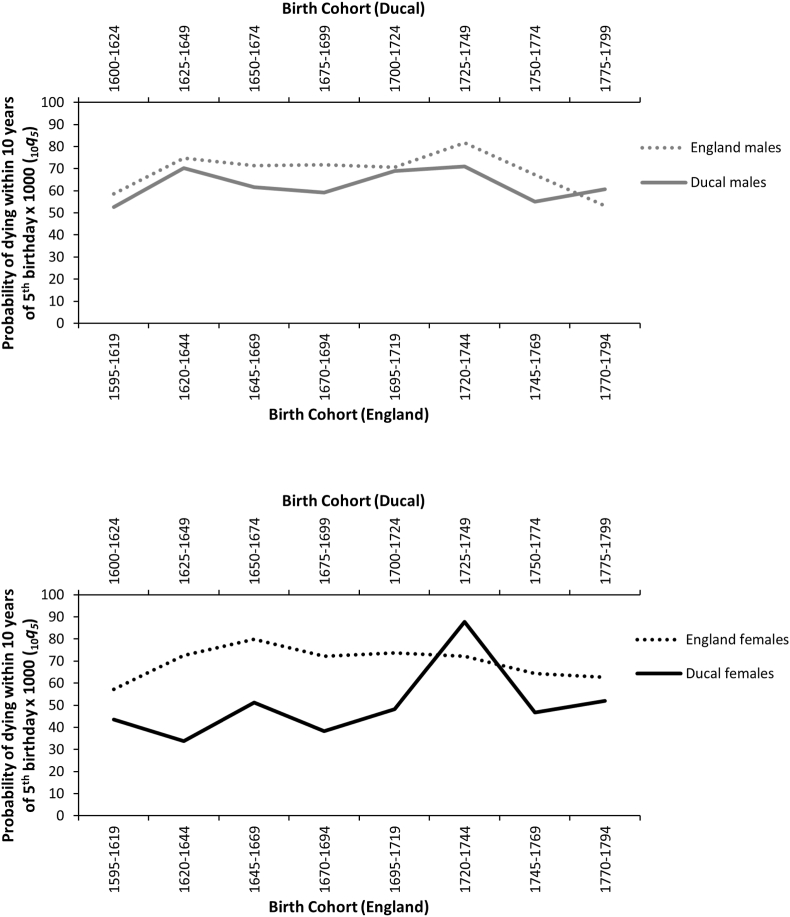
Probability of dying in childhood between the ages of 5 and 14 years during the 17^th^ and 18^th^ centuries for males (top panel) and females (bottom panel) in ducal (T. Hollingsworth, 1964, pp. 54–55) and English children (English data calculated from 5q5 and 5q10 in Wrigley et al. (1997), p. 296 using WHO method for CME (2013)).

Fig. 5 presents mortality trends for ages 5–14 in British aristocratic (Hollingsworth, 1964, pp. 54–55) and the wider English population by sex (English data calculated from 5q5 and 5q10 in Wrigley et al. (1997), p. 296 using WHO method for CME (2013)). A small but consistent advantage in mortality is evident for ducal males over the wider population. A larger gap is present favouring ducal females over the wider population, with the exception of a spike in mortality for the 1725–1749 female ducal birth cohort. Lower mortality in middle childhood and adolescence for ducal children of both sexes, relative to English children of the same periods, provides clear evidence of social inequality in mortality prior to 1750 for this age group.

## 4 Discussion

Taken together, these data suggest the existence of social inequality in health in all the time periods examined (i.e., from the 16^th^ century onwards). In analyses of life expectancy at birth, underlying inequality in health between the ducal and general population prior to 1750 is at least in part masked by the outsized effects of high under-5 mortality and, especially, high child mortality (1–4) among the aristocracy. Consanguineous marriages among the nobility, contracted in order to preserve status and wealth, must be considered as a factor in intrinsic childhood frailty; however, as these continued to be unremarkable, and indeed viewed as desirable, into the late 19^th^ century (Darwin, 1875), we must look for events or factors that changed substantially during the relevant period to explain the dramatic decreases in child mortality from the mid-18^th^ century. One such factor that might have contributed to high ducal childhood mortality prior to 1750 is the harmful effect of aristocratic child rearing practices in early modern England on vulnerability to disease – in particular, short birth spacing and early childhood feeding practices such as wet nursing or dry feeding.

One of the inherent privileges of wealth is the ability to follow fashions, whether in clothing, diet, or parenting behaviours, without being dictated to by necessity or privation. Fashionable behaviours may have positive or negative impacts on health. Where these are negative, they may obscure underlying health inequalities. In the case of the early modern English aristocracy, birth rates and rates of child mortality were high, both being largely driven by the longstanding tradition of using wet nurses and other servants to care for children. Exclusive maternal breastfeeding is suppressive of fertility, and this creates natural conditions for child spacing, which improves maternal health and reduces infant mortality (Norton, 2005; Thapa et al., 1988). Risks associated with short birth intervals (<36 months) and high parity (five or more pregnancies of 20 or more weeks gestation) are well-established and widely accepted to impact not only infant, but also child mortality, though mechanisms are contested (Conde-Agudelo et al., 2012; Kozuki & Walker, 2013). Common employment of wet nurses among the aristocracy led to annual pregnancies among aristocratic women through most of their childbearing years, with McLaren (2005) claiming that as many as 20 pregnancies was not uncommon among ultra-wealthy women of the 16^th^ to 18^th^ centuries, mirroring the trend towards high aristocratic parity in the medieval period (Podd, 2020). Sadly, few offspring survived childhood. Extreme parity among the aristocracy not only shortened the life expectancy of offspring but may also have decreased the lifespans of elite women, as Doblhammer and Oeppen (2003) found a trade-off between fertility and longevity in a re-evaluation of Hollingsworth's ducal data. Other scholars have linked longer birth intervals and lower child mortality among the common classes of England prior 1750 to higher prevalence of maternal breastfeeding (Davenport, 2019; McLaren, 1979).

By the 18^th^ century, English physicians had begun to take an interest in the care and medical treatment of infants. Physician William Cadogan's publication of a treatise advising parents of the importance of maternal breastfeeding to health made breastfeeding fashionable among the affluent and literate middling and upper classes, where infant and child mortality decreased sharply in the decades following publication (Fildes, 1980; Wickes, 1953a, 1953b). The importance of class and fashion to parenting practices and linked childhood health outcomes is also supported in bioarchaeological data on 19^th^ century London populations by Newman and Gowland (2017). Shifts in ducal child mortality data arising around 1750 are likely to be associated with the increasing fashion of maternal breastfeeding at that time, and linked improvements in maternal health. And, indeed, Hollingsworth (1957) relates a growth in the ducal population from the latter half of the 18^th^ century to an increase in offspring survival, rather than birth rate. If this is the case, then improvements in elite child survival were not the product of new health technologies, but the choice of the privileged to engage in a behaviour which had been long carried out by the poor through necessity.

The rise in overall life expectancy for ducal populations associated with a decrease in elite child mortality, together with data on status-linked advantages in later childhood and adolescent mortality, reveal what was likely always present: social inequality in health. Mortality risk for children between the ages of 5 and 14 years does not show a similar trend of improvement over time to early childhood, in either the ducal or wider English data. There is no reduction in risk associated with the mid to late 18^th^ century for older ducal children, which might be expected if — as proposed in previous studies — gains in life expectancy were enabled by emerging medical innovations only available to the wealthy from 1750. Instead, the trends in middle childhood and adolescent mortality risk remain largely unchanged between the 17^th^ and 19^th^ centuries for both ducal and non-ducal children, with lower risk of death for ducal children being a reasonably consistent feature of that period, regardless of sex. Existing advantages connected to environment and resources, rather than novel medical advances, are a more parsimonious explanation for this underlying disparity. Throughout life, the wealthy were protected from many of the threats to health which were a daily part of life for the general population, including poor indoor air quality, overcrowding and poor sanitation, psychosocial stress, undernutrition, violent crime, occupational hazards, and the ability to relocate during periods of epidemic disease (Gowland, 2018; Newman, 2012; Newman, 2021). These differences in life experience would have created disparities in vulnerability to infectious or chronic diseases for elites and common people. Importantly, mortality data does not address socially mediated differences in morbidity, which are likely to have been considerable. However, based on childhood and adolescent mortality data, we suggest that underlying health inequalities predate 1750, and that life expectancy at birth may not be a reliable indicator of health inequalities in all periods.

## 5 Conclusions

Prior to 1750, high child mortality rates in ducal families may be linked to the use of wet nurses, resulting in narrow birth intervals and extreme fertility. High child mortality may have masked underlying social inequality in health in measures such as life expectancy from birth. The rise in life expectancy among ducal families from 1750 coincides with changes to elite childrearing practices and reductions in child mortality, which provide important context for interpreting mortality patterns. We also find evidence of a consistent gap in mortality for ages 5–14 between aristocratic and non-aristocratic families, both before and after 1750. Social inequalities in mortality appear to have existed before 1750 but varied with age; there were inequalities favouring the general population for under-5 mortality but inequalities favouring the ducal class for age 5–14 mortality. We therefore conclude that health inequality existed well before 1750; however, the impacts of social inequalities in health are mediated by age.

## Author statements

Ellen Kendall: Conceptualization, Data curation, Formal analysis, Investigation, Methodology, Validation, Visualization, Writing - original draft, Writing - review & editing; Alex Brown: Conceptualization, Methodology, Supervision, Writing - review & editing; Tim Doran: Conceptualization, Writing - Review & Editing, Supervision, Funding acquisition; Rebecca Gowland: Conceptualization, Writing - Review & Editing, Supervision; Richard Cookson: Conceptualization, Writing - Original Draft, Writing - Review & Editing, Supervision, Project administration.

## Ethics statement

The authors declare that there are no financial or personal relationships with other people or organizations that could have inappropriately influenced or biased their work.

## Declaration of competing interest

The authors declare that they have no known competing financial interests or personal relationships that could have appeared to influence the work reported in this paper.
